# Stereological and biochemical effects of thymoquinone on ovarian tissue toxicity induced by silver nanoparticles in NMRI mice: An experimental study

**DOI:** 10.18502/ijrm.v22i7.16964

**Published:** 2024-09-12

**Authors:** Seyed Mohammad Ali Shariatzadeh, Fatemeh Salmani, Hossein Moghanlo, Monireh Mahmoodi

**Affiliations:** Department of Biology, Faculty of Sciences, Arak University, Arak, Iran.

**Keywords:** Nanoparticles, Silver, Thymoquinone, Ovary, Mouse.

## Abstract

**Background:**

The toxicity of silver nanoparticles (AgNPs) has been proven in the female reproductive system. Thymoquinone (TQ) is a natural antioxidant and bioactive component of *Nigella sativa*.

**Objective:**

We evaluated the efficacy of TQ on ovarian tissue following toxicity induced by AgNPs in female mice.

**Materials and Methods:**

24 female NMRI mice (5–6 wk, an average weight of 33 gr) were randomly divided into 4 groups (n = 6/each): control, AgNPs (500 mg/kg, gavage), TQ (2.5 mg/kg, intraperitoneal injection), and TQ+AgNPs. Mice were treated every day for 35 days. Serum levels of malondialdehyde (MDA), total antioxidant capacity (TAC), luteinizing hormone, and follicle-stimulating hormone were measured. The optical disector and stereological techniques were utilized to estimate the follicular count, their volume at different developmental stages, and the structure of ovarian tissue.

**Results:**

In the AgNPs group, the serum concentrations of TAC (p = 0.01), luteinizing hormone (p < 0.001), follicle-stimulating hormone, the volume of corpus luteum (p < 0.001), and the number of follicles decreased significantly compared to the control group. Nevertheless, AgNPs significantly increased the MDA level. In the TQ+AgNPs group compared to the AgNPs group, a significant decrease in MDA level (p < 0.001) and a significant improvement in TAC (p = 0.03), and hormonal levels, the number of primary, preantral, and antral follicles (p = 0.04), and the volume of corpus luteum (p = 0.01) were observed.

**Conclusion:**

TQ improved the number of follicles by reducing oxidative stress and lipid peroxidation in AgNPs-damaged ovarian tissue.

## 1. Introduction

Nowadays, several factors, such as changing lifestyles, exposure to environmental toxins, underlying medical conditions, and genetic predispositions, have impacted couples' fertility (1). The specific biological properties of silver nanoparticles (AgNPs) have led to their widespread use in various industries, such as medicine, plastics, textiles, water refineries, air filters, and agriculture (2). In medical science, AgNPs are used for wound dressing, skin ointments, disinfectants, drug delivery systems, and dentistry due to their antiviral, antibacterial, and antifungal properties (3). Despite these benefits, many studies show that long-term exposure to AgNPs induces cellular and tissue toxicity in various tissues and reproductive organs (4, 5). AgNPs enter cells by crossing blood barriers and lead to cytotoxicity due to damage to cell organelles, especially the mitochondrial electron transport chain (6). The reproductive organs are vulnerable to AgNPs in a dose- and time-dependent manner (7).

Regarding the female reproductive system, AgNPs in different sizes, depending on the dose, cause severe damage to ovarian tissue and follicular cells (8). Currently, laboratory studies have demonstrated that AgNPs can cause developmental toxicities in the fetus (9). Exposure to AgNPs by disrupting the process of aromatization and steroidogenesis causes a decrease in estrogen, degeneration of follicles, and induction of apoptosis in ovarian cells (10). AgNPs have toxic effects on the development of mouse embryos; histopathological studies have shown that the toxic dose of AgNPs passes through the placenta, causing a decrease in cell viability and damaging the follicular cells in the ovarian tissue (11). Through the induction of lipid peroxidation and reactive oxygen species, AgNPs decreased Cyp19a1 enzyme (cytochrome P450, family 19, subfamily a, polypeptide 1) activity, estrogen levels, and destruction of preantral and antral follicles (12).

Herbal medicines are of particular importance as alternative medicines. They have reduced the concern caused by the side effects of synthetic drugs. One of the effective compounds is thymoquinone (TQ). TQ (2-isopropyl-5-methylbenzo-1,4-quinone), as a natural antioxidant, is a bioactive ingredient of *Nigella sativa *(13). Molecular signaling pathways show that TQ protects cells against oxidative stress by regulating the activity of antioxidant enzymes. They can also play an essential role in cell survival or apoptosis by regulating inflammatory cytokines and factors involved in cell cycle regulation (14). The pharmacological effects of TQ, including anti-inflammatory, modulation of the immune system, and anticancer effects, have been proven in previous studies. In addition, TQ improves cardiac, neurological (neurological inflammation, Alzheimer's and Parkinson's diseases), respiratory, digestive, liver, kidney, and other disorders. Besides, it is very effective in antiobesity and antidiabetes (15). Previous studies have shown the therapeutic effect of TQ on the male and female reproductive system (16). Recent findings have shown that TQ has a protective impact on polycystic ovary syndrome (17).

Based on recent searches, there was no stereological analyses of the effects of TQ on the toxic effects of AgNPs on mouse ovarian structure. The present study evaluated the effectiveness of TQ against the toxicity induced by AgNPs on ovarian tissue in mice. The stereological analysis of ovarian tissue, the follicular count and their volume at different developmental stages, the measurement of oxidative stress indices, and the level of sex hormones evaluated.

## 2. Materials and Methods

### Preparation of chemicals

The commercial AgNPs (US Research Nanomaterials, Inc., USA) were purchased from Pishgaman Nanomaterials Co., Iran. The authenticity of the quality of AgNPs (spherical morphology, 99.99% purity, average diameter 20 nm, metal base) was evaluated and confirmed through X-ray diffraction and transmission electron microscopy by Pishgaman Nanomaterials Co., Iran. Also, TQ with characteristics (2-Isopropyl-5-methyl-1, 4-benzoquinone, ≥ 98.0%, molecular formula: C ${\;}_{\text{10}}$ H ${\;}_{\text{12}}$ O ${\;}_{\text{2}}$ , molecular weight: 164.20 g.mol ${\;}^{-1}$ ) (Sigma-Aldrich, USA) was purchased.

### Sample size

According to previous studies, alpha error was considered 5% (4, 15). Subsequently, the sample size was determined using the N = DF/K + 1 formula, where N, DF, and K denote the number of mice per group, degrees of freedom, and the number of groups, respectively. Therefore, 24 mice (n = 6/each) were utilized for the experiment.

### Experimental design and mice

In this experiment, 24 healthy 5–6 wk-old NMRI female mice with an average weight of 33 ± 1.5 gr were used. Mice were purchased from the Pasteur Institute (Tehran, Iran). 1 wk before the beginning of the experiment, the animals were kept in the animal house of Arak University under standard conditions for 12 hr light/dark cycle at a temperature of 21 ± 2 C with free access to a standard diet and water. We closely monitored the mice's nutritional behavior, body weight, behavior, and physical appearance changes during the treatment period. Therefore, we committed to halting the treatment immediately if we noticed any signs of suffering.

Mice were randomly divided into 4 groups (n = 6/each): control group, AgNPs group (500 mg/kg) (4, 7), TQ group (2.5 mg/kg) (15), and co-administration of AgNPs + TQ group (2.5 mg/kg TQ + 500 mg/kg AgNPs). According to the average weight of female mice (33 gr), solutions of 500 mg/kg bwt AgNPs in distilled water and 2.5 mg/kg bwt TQ in dimethyl sulfoxide were prepared daily. Thus, mice were treated with AgNPs orally (via gavage tube) and TQ via intraperitoneal injection for 35 consecutive days (12). At the end of the treatment period, the percentage of changes in the body weight of mice was calculated using the formula (W ${\;}_{\text{2}}$ -W ${\;}_{\text{1}}$ ) / W ${\;}_{\text{1}}\times$ 100, where W ${\;}_{\text{1}}$ is the initial body weight of the mouse at the beginning of the experiment, and W ${\;}_{\text{2}}$ is the final body weight at the end of the experiment. Mice were anesthetized using the 10 mg/kg intraperitoneal injections of xylazine (2%, Alfasan, Netherlands) and the 100 mg/kg ketamine (10%, Alfasan, Netherlands), after which blood was collected from the heart, serum samples were obtained via centrifugation (13,000 rpm, 10 min). The serum samples were frozen at -80 C for biochemical and hormonal assay. The left and right ovaries were removed, the right ovaries were weighed to determine the weight change of the ovary, and the left ovaries were fixed in Bouin's fixative solution for 24 hr for tissue processing and stereological techniques (18).

### Tissue processing and stereological analysis

Tissue processing was performed via an automatic processor (Histokinette, Leica, Germany). In brief, spherical and cylindrical tissue blocks were obtained from the paraffin dispenser (DS-4L, Didsabz, Iran). Sections were prepared using the isotropic uniform random method and a Leitz 1512 rotary microtome (Leitz, USA). For stereological studies, 10–12 sections per ovary are required (18). Approximately 120 sections (i.e., 60 sections of 5-micron and 60 sections of 20-micron) alternately were obtained, per ovary. Subsequently, using the systematic sampling and K = N/n formula, 20 sections were selected for each ovary (i.e., 10 sections of 20-micron and 10 sections of 5-micron per ovary) to the hematoxylin and eosin staining. K, N, and n denote the uniform distance between 2 samples, the total number of samples, and the number of samples, respectively.

#### Estimation of volume (ovary, medulla, cortex, and corpus luteum)

**Figure 1:**
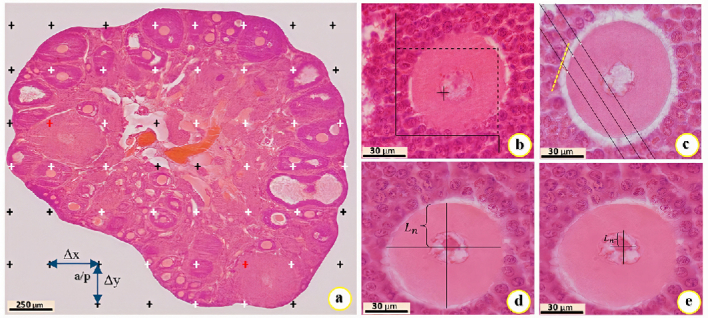
A) The cavalieri technique to estimate the volume. B) The unbiased counting frame to estimate the follicular count. C) The harmonic method to estimate the thickness of zona pellucida. D, E) The nucleator method to estimate the volume of the oocyte and its nucleus. a/p and Δ x, Δ y represent the area and distance between 2 points in the x-y axis, respectively.

The Cavalieri technique and 10 sections of 5-micron per ovary were employed to estimate the volume of ovarian structure. Briefly, an optical microscope (BX41, Olympus, Japan) equipped with a camera (DP12, Olympus, Japan) and the images of ovarian tissue were reflected on the monitor with × 40 magnification. The point probe was randomly dropped on the images. In each ovary, the total number of collision points with all images of the sections and 200 collision points for each section of the cortex, medulla, and corpus luteum were counted. Based on the following formulas, ` ∑ p' is the total collision points between the probe and images, `a(p)' is the area of each point on the probe, `t' is the thickness of the sections, ` Δ x' and ` Δ y' are the distance between 2 points (Figure 1a), `M' denotes the microscope magnification, ` ∑ p ${\;}_{\text{medulla}}$ ' is probe collisions with the medulla, ` ∑ p ${\;}_{\text{cortex}}$ ' is probe collisions with the cortex, ` ∑ p ${\;}_{\text{corpus}}{\;}_{\text{luteum}}$ ' denotes the probe collisions with the corpus luteum (18). 


$$V_{\text{total ovary}}={\sum p}_{\text{total}}\times a(p)\times t$$

$$a(p)=\left(\Delta x\times \Delta y\right)/M^{2}$$

$$V_{\text{medulla}}=[{\sum p}_{\text{medulla}}/{\sum p}_{\text{total}}]\times [V_{\text{total ovary}}]$$

$$V_{\text{cortex}}=\left[{\sum p}_{\text{cortex}}/{\sum p}_{\text{total}}]\times [V_{\text{total ovary}}\right]$$

$$V_{\text{corpus luteum}}=\left[{\sum p}_{\text{corpus luteum}}/{\sum p}_{\text{cortex}}]\times [V_{\text{cortex}}\right]$$

#### Estimation of the number of follicles

The optical disector method and optical microscope (BX41, Olympus, Japan) equipped with a microcator device (ND221B, Heidenhain, Germany) were used to estimate the follicular count at different developmental stages. The images of 20-micron sections were reflected on the monitor with × 1000 magnification. The number of cells at a depth of 10 µm was estimated using a microcator and an unbiased counting frame (Figure 1b). 5 µm from the top and bottom of the 20 µm sections as guard zones were omitted. 100–150 cells were counted for each ovary. According to the following formula, the number of follicles, including primordial, primary, preantral, and antral were estimated in ovarian tissue. Where ` ∑ Qi', ` ∑ p', and `a/f' denote the number of cells counted, the total number of counting frames that hit the fields of view, and the area of the unbiased counting frame, respectively. Also, `h' is the height of the disector which was 10 μm, and `V ${\;}_{\text{total ovary}}$ ' represents the total volume of the ovary calculated by the Cavalieri method (18). 


$$N_{V}=\left[\sum Qi/\left(h\times \sum p\times a/f\right)]\times [V_{\text{total ovary}}\right]$$

#### Estimation of zona pellucida thickness in the preantral and antral follicles

At first, microscopic images from 5-micron sections were randomly selected, and the harmonic method was used to measure the thickness of the zona pellucida. Then, a special probe with 3 parallel and equal lines was randomly dropped on the microscopic images with × 1000 magnification (Figure 1c). In this method, from the intersection of probe lines with the inner surface of the zona pellucida, a vertical line was drawn to the tangent line of the outer surface of the zona pellucida. The length of the vertical line (orthogonal intercept) was measured by Motic Images software. An average of 120 orthogonal intercepts (oi ${\;}_{\text{1}}$ , oi ${\;}_{\text{2}}$ , oi ${\;}_{\text{3}}$ , ...., oi ${\;}_{\text{119}}$ , oi ${\;}_{\text{120}}$ ) were measured for each ovary. Finally, the mean thickness of the zona pellucida (ZP) was estimated using the following formula: Where `N' represents the total number of measurements, and `oi' shows the length of each vertical line from the inner surface to the outer surface of the zona pellucida (18) (π = 3.14). 


$$ThicknessofZP=N/[1/{oi}_{1}+1/{oi}_{2}+\cdots ]\times 8\pi /3$$

#### Estimation of the volume of the oocyte and its nucleus

According to the nucleator method, images of 20-micron sections were randomly selected using an optical microscope at × 1000 magnifications (BX41, Olympus, Japan), equipped with a camera (DP12, Olympus, Japan) and an unbiased counting frame. In brief, probing in the depth of sections, the oocyte's nucleus in maximum focus was selected (if the nucleus is not apparent, the center of the nucleus was considered as the virtual center of the nucleus) (18). 2 straight lines were drawn in different directions from the nucleus's center to the nuclear membrane (to estimate the volume of the nucleus) and to the oocyte membrane (to estimate the volume of the oocyte) (Figure 1d, e). Finally, the following formula was used, where `V ${\;}_{\text{n}}$ ' represents the volume of the oocyte or nucleus and `Ln' displays the distance from the nucleus's center to the nucleus membrane or from the nucleus center to the oocyte membrane. The results were reported as µm^3^ ( π = 3.14). 


$$V_{\text{n}}=4\pi /3\times \bar{L_{\text{n}}^{3}}$$

### Lipid peroxidation assay

Lipid peroxidation in the damaged cell membrane increases the production of malondialdehyde (MDA). Therefore, MDA was measured as an index of lipid peroxidation. MDA assay was performed via the Aust and Buege method. Briefly, a reagent solution, including 0.375% thiobarbituric acid (Merck, Germany), 15% trichloroacetic acid (Merck, Germany), and 0.25 N hydrochloric acid. 200 μl of reagent and 100 μl of serum were mixed and placed in a bain-marie at 95 C for 15 min, then cooled and centrifuged (10 min, 1000 rpm). Lastly, the supernatant solution obtained was measured at 532 nm by spectrophotometer (PG Instruments Ltd, UK). Finally, the MDA level was calculated using the extinction coefficient of 1.56 × 10^5^ M ${\;}^{-1}$ cm ${\;}^{-1}$ (5).

### Ferric reducing antioxidant power (FRAP) assay

The FRAP method was applied to measure the total antioxidant capacity (TAC) (FRAP method presented by Benzie and Strain). Briefly, the FRAP reagent was prepared in a 1:1:10 ratio of 20 mM ferric chloride, 10 mM 2,4,6-tri(2-pyridyl)-s-triazine (TPTZ; Sigma-Aldrich, USA), and 300 mM sodium acetate buffer (pH 3.6). Also, different concentrations of FeSO4.7H2O were prepared. 1.5 ml of FRAP reagent was added to 50 μl of serum sample and FeSO4.7H2O. It was then incubated for 4 min at 37 C bain-marie, and absorbance at a wavelength of 593 nm by a spectrophotometer (PG Instruments Ltd, UK) was measured. The standard curve of different concentrations of FeSO4.7H2O was drawn using GraphPad Prism software. Finally, TAC levels were calculated in serum samples (5).

### Hormonal assay

The levels of luteinizing hormone (LH) and follicle-stimulating hormone (FSH) in the experimental groups were measured by the enzyme-linked immunosorbent assay (ELISA, Pishtazteb, Iran). Based on the kit protocol, hormonal levels were measured by sandwich ELISA method and using the monoclonal antibodies. Finally, the concentration of hormones was reported as IU/L.

### Ethical considerations 

According to the principles approved by the Research Ethics Committee of Arak University, Arak, Iran (Code: IR.ARAKU.REC.1400.003), laboratory mice were employed in this study. All procedures were carried out following the ethical and ARRIVE guidelines.

### Statistical analysis

Data were analyzed using the SPSS statistics software (Version 26, IBM Inc., USA). Shapiro-Wilk and Kolmogorov-Smirnov statistical tests were used to evaluate the normality of the data. Finally, considering the significance level p < 0.05 and 95% confidence interval, data were analyzed using one-way analysis of variance (ANOVA) and Tukey's post hoc test.

## 3. Results

In this experimental study, all 24 healthy 5–6 wk-old NMRI female mice (Pasteur Institute, Tehran, Iran) were treated in 4 groups until the end of the treatment period for 35 days. Hence, all participants data were reported.

### Body and right ovary weight

No significant changes were observed in the body and ovary weight in the AgNPs group compared to the control group. Also, no difference was observed between the experimental groups. The detailed data are shown in table I.

### Histological findings

**Figure 2:**
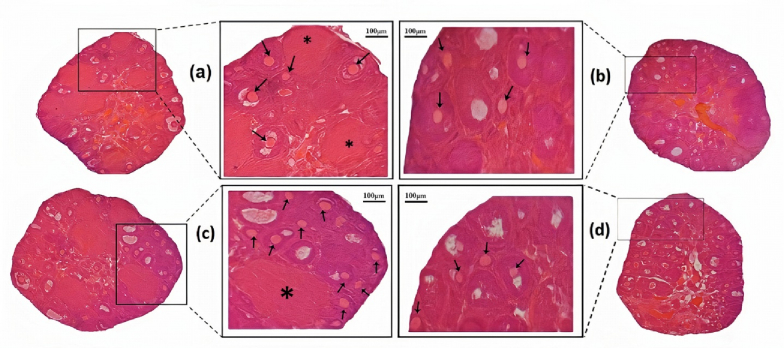
Microscopic observations of mice ovary after 5 wk of treatment. A) Control group. B) AgNPs group: The different types of follicles were decreased. C) TQ group. D) TQ+AgNPs group. The number of follicles was increased compared to the AgNPs group. Scale bar: 100 μm, hematoxylin-eosin staining (40-fold magnification). Black arrow: The different types of follicles, Asterisk (*): Corpus luteum.

Based on microscopic studies (Figure 2), folliculogenesis, the number of follicles, and corpus luteum in control and TQ groups were normal. In contrast, fewer follicles were observed in the AgNPs group. Meanwhile, the different types of follicles were observed in the co-administration group (TQ+AgNPs), similar to the control group.

### Volume of the ovary, medulla, cortex, and corpus luteum

No differences were observed in the mean total volume of the ovary, medulla, and cortex in the AgNPs group compared to the control group. In contrast, an increase in the mean total volume of the ovary, medulla, and cortex in the TQ group compared to the AgNPs group, were observed (Table II). However, AgNPs caused a decrease in the volume of the corpus luteum in the AgNPs group compared to the control group. On the other hand, the volume of corpus luteum in the co-administration group (TQ+AgNPs) was improved compared to the AgNPs group, so it was observed at the level of the control group. Also, an increase in corpus luteum volume was observed in the TQ group compared to the control group (Table II).

### Volume of the oocyte and its nucleus, and thickness of zona pellucida

Compared to the control group, no differences were observed in the mean volume of oocytes in primary, primordial, preantral, and antral follicles in the AgNPs group (Table II). Compared to the control group, no differences were observed in the mean volume of the oocyte nucleus in primary, primordial, preantral, and antral follicles in the AgNPs group (Table III). Also, no difference was observed in the mean thickness of the zona pellucida in preantral and antral follicles in the AgNPs group when compared with the control group (Table III).

### Follicular count 

A decrease in the number of primordial, primary, preantral, and antral follicles was seen in the AgNPs group compared to the control group. In the TQ+AgNPs group, an increase in the number of primary, preantral, and antral follicles was observed compared to the AgNPs group. Although the number of primordial follicles in the TQ+AgNPs group improved compared to the AgNPs group, this increase was insignificant. In addition, an increase in the number of primary, preantral, and antral follicles was observed in the TQ group compared to the control group (Table IV).

### FRAP and lipid peroxidation

**Figure 3:**
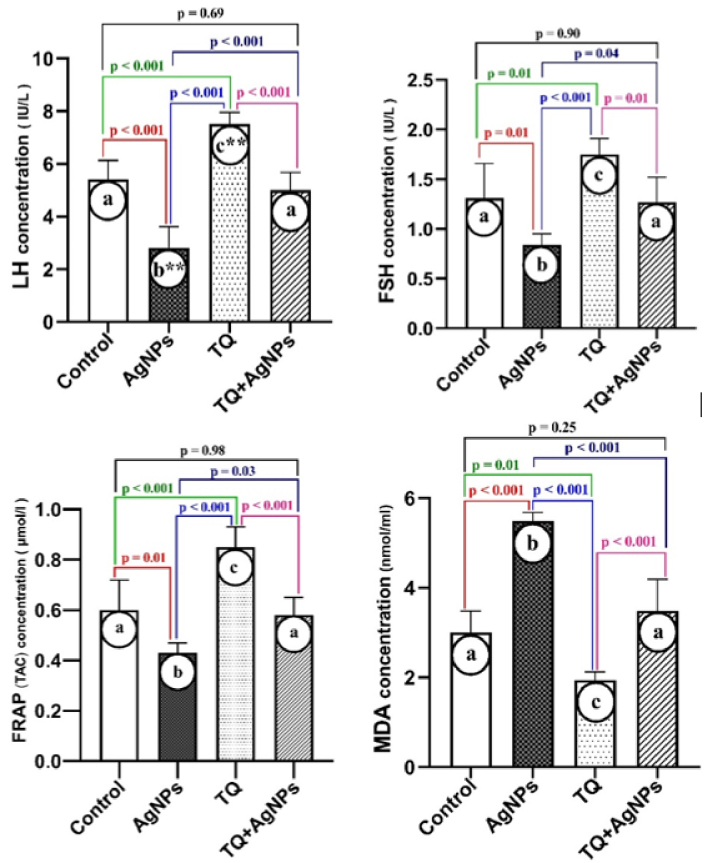
The oxidative stress indices and hormonal assay in the female mice. Data were expressed as Mean ± SD (n = 6), ANOVA, and Tukey's post hoc test. The different superscripts (a-c) represent significant differences within means at p < 0.05. TQ: Thymoquinone, AgNPs: Silver nanoparticles, MDA: Malondialdehyde, TAC: Total antioxidant capacity, FRAP: Ferric reducing antioxidant power, LH: Luteinizing hormone, FSH: Follicle-stimulating hormone.

As shown in figure 3, the results of oxidative stress indices showed that the level of MDA increased in the AgNPs group. However, it was decreased in the co-administration group (TQ + AgNPs) compared to the AgNPs group. In addition, a decrease in MDA was observed in the TQ group compared to the control group. Also, the results of FRAP showed a decrease in TAC in the AgNPs group compared to the control group. In contrast, the level of TAC in the TQ + AgNPs group was increased compared to the AgNPs group.

### The sexual hormones levels: LH and FSH

As shown in figure 3, compared to the control group, the levels of LH and FSH hormones were decreased in the AgNPs group. In parallel, in the TQ + AgNPs group, improvement of LH and FSH were observed compared to the AgNPs group. Also, TQ increased the concentration of LH and FSH compared to the control group.

**Table 1:** Effect of TQ and AgNPs administration on the percentage of body weight changes and ovary weight in the female mice


**Parameters**	**Control**	**AgNPs**	**TQ**	**TQ + AgNPs**	**P-value**
Initial body weight (gr)*	31.16 ± 0.69 (31.25, 1.18)	31.61 ± 1.22 (32.35, 2.55)	30.98 ± 0.90 (30.90, 1.68)	31.48 ± 1.62 (30.65, 3.20)	a = 0.66 b = 0.99 c = 0.96 d = 0.50 e = 0.90 f = 0.88
Final body weight (gr)*	31.76 ± 0.79 (31.45, 1.47)	31.18 ± 1.15 (31.35, 2.00)	31.33 ± 0.67 (31.20, 1.25)	31.06 ± 0.46 (30.90, 0.9)	a = 0.61 b = 0.79 c = 0.46 d = 0.98 e = 0.99 f = 0.94
Percentage of body weight changes (%)**	1.92 (2.38, 2.93)	-1.35 (-1.43, 2.07)	1.13 (1.13, 1.65)	-1.17 (0.49, 6.85)	a = 0.08 b = 0.92 c = 0.10 d = 0.24 e = 0.99 f = 0.30
Right ovary weight (gr)*	0.023 ± 0.002 (0.023, 0.005)	0.020 ± 0.004 (0.022, 0.007)	0.022 ± 0.003 (0.022, 0.007)	0.021 ± 0.002 (0.021, 0.003)	a = 0.65 b = 0.95 c = 0.85 d = 0.92 e = 0.98 f = 0.99
*Data are presented as Mean ± SD (MD, IQR), ANOVA, and Tukey's post hoc test. **Data are presented as percentages (MD, IQR), ANOVA, and Tukey's post hoc test. The different scripts show P-value within means in each row. a: Control vs AgNPs, b: Control vs TQ, c: Control vs TQ + AgNPs, d: AgNPs vs TQ, e: AgNPs vs TQ + AgNPs, f: TQ + AgNPs vs TQ, TQ: Thymoquinone, AgNPs: Silver nanoparticles					

**Table 2:** Effect of TQ and AgNPs administration on the volume of ovary, medulla, cortex, corpus luteum, and mean volume of oocytes at different developmental stages in the female mice


**Parameters**	**Control**	**AgNPs**	**TQ**	**TQ + AgNPs**	**P-value**
**Volume of ovary (mm^3^)**	2.27 ± 0.34 ${\;}^{*†}$ (2.17, 0.62)	1.90 ± 0.16 ${\;}^{*}$ (1.92, 0.31)	2.70 ± 0.27 ** ${\;}^{†}$ ** (2.74, 0.56)	2.14 ± 0.30 ${\;}^{*}$ (2.12, 0.53)	a = 0.12 b = 0.06 c = 0.86 d ≤ 0.001 e = 0.44 f = 0.01
**Volume of medulla (mm^3^)**	0.50 ± 0.04 ${\;}^{*}$ (0.50, 0.09)	0.44 ± 0.04 ${\;}^{*}$ (0.43, 0.07)	0.62 ± 0.10 ** ${\;}^{†}$ ** (0.61, 0.17)	0.43 ± 0.08 ${\;}^{*}$ (0.43, 0.15)	a = 0.66 b = 0.04 c = 0.55 d = 0.01 e = 0.99 f = 0.01
**Volume of cortex (mm^3^)**	1.77 ± 0.30 ${\;}^{*†}$ (1.66, 0.54)	1.46 ± 0.16 ${\;}^{*}$ (1.45, 0.29)	2.09 ± 0.18 ** ${\;}^{†}$ ** (2.13, 0.34)	1.71 ± 0.27 ${\;}^{*†}$ (1.71, 0.44)	a = 0.13 b = 0.12 c = 0.97 d = 0.01 e = 0.28 f = 0.06
**Volume of corpus luteum (mm^3^)**	0.46 ± 0.06 ${\;}^{*}$ (0.48, 0.12)	0.27 ± 0.04 ** ${\;}^{†}$ ** (0.26, 0.07)	0.57 ± 0.06 ** ${\;}^{\#}$ ** (0.57, 0.10)	0.39 ± 0.05 ${\;}^{*}$ (0.40, 0.09)	a ≤ 0.001 b = 0.03 c = 0.17 d ≤ 0.001 e = 0.01 f ≤ 0.001
**Oocyte volume of primordial follicles (μm^3^)**	1647.36 ± 220.59 ${\;}^{*†}$ (1553.29, 397.42)	1331.71 ± 162.99 ${\;}^{*}$ (1349.24, 305.82)	1707.89 ± 249.73 ** ${\;}^{†}$ ** (1714.62, 435.82)	1433.93 ± 191.01 ${\;}^{*†}$ (1442.11, 363.80)	a = 0.07 b = 0.96 c = 0.32 d = 0.02 e = 0.83 f = 1.40
**Oocyte volume of primary follicles (μm^3^)**	6218.42 ± 1130.57 ${\;}^{*†}$ (6318.79, 2212.24)	5101.55 ± 171.67 ${\;}^{*}$ (5078.95, 297.58)	6965.04 ± 993.89 ** ${\;}^{†}$ ** (6912.48, 1990.98)	5181.66 ± 356.71 ${\;}^{*}$ (5295.99, 495.32)	a = 0.09 b = 0.37 c = 1.29 d = 0.01 e = 0.99 f = 0.01
**Oocyte volume of preantral follicles (μm^3^)**	60133.59 ± 9370.39 ${\;}^{*†}$ (59524.36, 18132.93)	50627.61 ± 1272.64 ${\;}^{*}$ (50674.58, 2265.89)	63897.00 ± 8414.59 ** ${\;}^{†}$ ** (64790.92, 14445.33)	55330.06 ± 2491.38 ${\;}^{*†}$ (54927.41, 4705.50)	a = 0.08 b = 0.74 c = 0.58 d = 0.01 e = 0.59 f = 0.13
**Oocyte volume of antral follicles (μm^3^)**	144595.14 ± 9301.81 ${\;}^{*†}$ (143711.48, 16374.56)	128417.22 ± 8574.46 ${\;}^{*}$ (126870.83, 15992.47)	160675.76 ± 16871.50 ** ${\;}^{†}$ ** (166302.29, 25825.09)	132618.11 ± 4882.22 ${\;}^{*}$ (131930.91, 8398.11)	a = 0.08 b = 0.08 c = 0.25 d ≤ 0.001 e = 0.91 f = 0.01
Data are presented as Mean ± SD (MD, IQR), ANOVA, and Tukey's post hoc test. The superscripts (*, † , and #) show significant differences within means at p < 0.05 in the same row. The different scripts show P-value within means in each row. a: Control vs AgNPs, b: Control vs TQ, c: Control vs TQ + AgNPs, d: AgNPs vs TQ, e: AgNPs vs TQ + AgNPs, f: TQ + AgNPs vs TQ, TQ: Thymoquinone, AgNPs: Silver nanoparticles					

**Table 3:** Effect of TQ and AgNPs exposure on the mean volume of oocyte's nucleus and thickness of zona pellucida in preantral and antral follicles


	**Parameters**	**Control**	**AgNPs**	**TQ**	**TQ + AgNPs**	**P-value**
**The volume of the oocyte's nucleus**						
	**Oocyte's nucleus** **volume of primordial follicles (μm^3^)**	512.80 ± 82.69 ${\;}^{*†}$ (520.59, 122.58)	422.13 ± 42.21 ${\;}^{*}$ (422.37, 72.70)	548.42 ± 37.64 ** ${\;}^{†}$ ** (553.53, 67.04)	432.27 ± 53.07 ${\;}^{*}$ (416.91, 90.52)	a = 0.06 b = 0.70 c = 0.09 d = 0.01 e = 0.98 f = 0.01
	**Oocyte's nucleus** **volume of primary follicles (μm^3^)**	1364.64 ± 123.58 ${\;}^{*†}$ (1387.48, 221.87)	1201.23 ± 72.00 ${\;}^{*}$ (1214.69, 108.83)	1496.11 ± 79.65 ** ${\;}^{†}$ ** (1498.67, 145.49)	1229.03 ± 122.75 ${\;}^{*}$ (1214.83, 246.84)	a = 0.06 b = 1.50 c = 0.13 d ≤ 0.001 e = 0.96 f = 0.01
**The volume of the oocyte's nucleus**						
	**Oocyte's nucleus** **volume of preantral follicles (μm^3^)**	2216.40 ± 225.68 ${\;}^{*†}$ (2249.94, 426.79)	1902.99 ± 165.97 ${\;}^{*}$ (1901.71, 324.50)	2375.60 ± 259.31 ** ${\;}^{†}$ ** (2338.28, 411.26)	2088.35 ± 233.59 ${\;}^{*†}$ (2025.33, 472.27)	a = 0.10 b = 0.61 c = 0.75 d =0.01 e = 0.49 f = 0.15
	**Oocyte's nucleus** **volume of antral follicles (μm^3^)**	4916.31 ± 291.67 ${\;}^{*†}$ (4958.33, 521.73)	4584.90 ± 303.81 ${\;}^{*}$ (4562.20, 594.21)	5190.68 ± 177.74 ** ${\;}^{†}$ ** (5201.20, 344.10)	4680.79 ± 111.60 ${\;}^{*}$ (4668.84, 206.39)	a = 0.10 b = 0.21 c = 0.33 d = 0.01 e = 0.89 f = 0.01
**Thickness of zona pellucida**						
	**Preantral follicles (μm) **	11.85 ± 0.30 ${\;}^{*†}$ (11.90, 0.62)	11.41 ± 0.56 ${\;}^{*†}$ (11.59, 1.08)	12.03 ± 0.24 ** ${\;}^{†}$ ** (11.99, 0.44)	11.31 ± 0.43 ${\;}^{*}$ (11.34, 0.81)	a = 0.26 b = 0.87 c = 1.27 d = 0.06 e = 0.97 f = 0.03
	**Antral follicles (μm)**	16.86 ± 0.42 (16.91, 0.59)	16.56 ± 0.50 (16.60, 0.93)	17.06 ± 0.62 (17.02, 1.12)	16.68 ± 0.62 (16.71, 1.10)	a = 0.78 b = 0.92 c = 0.94 d = 0.41 e = 0.98 f = 0.62
Data are presented as Mean ± SD (MD, IQR), ANOVA and Tukey's post hoc test. The superscripts (* and † ) show significant differences within means at p < 0.05 in the same row. The different scripts show P-value within means in each row. a: Control vs AgNPs, b: Control vs TQ, c: Control vs TQ + AgNPs, d: AgNPs vs TQ, e: AgNPs vs TQ + AgNPs, f: TQ + AgNPs vs TQ, TQ: Thymoquinone, AgNPs: Silver nanoparticles						

**Table 4:** Effect of TQ4 and AgNPs on the mean number of types of follicles in the female mice


**Parameters**	**Control**	**AgNPs**	**TQ**	**TQ + AgNPs**	**P-value**
**Primordial follicles**	2125.18 ± 192.54 ${\;}^{*\#}$ (2098.00, 287.68)	1847.45 ± 187.12 ** ${\;}^{†}$ ** (1762.81, 340.06)	2397.72 ± 156.83 ** ${\;}^{\#}$ ** (2366.78, 6030.71)	2042.09 ± 138.79 ${\;}^{*†}$ (2030.01, 257.38)	a = 0.04 b = 0.53 c = 0.83 d ≤ 0.001 e = 0.23 f = 0.01
**Primary follicles**	692.40 ± 49.57 ${\;}^{*}$ (713.72, 67.23)	606.60 ± 48.35 ** ${\;}^{†}$ ** (597.80, 79.86)	787.31 ± 15.25 ** ${\;}^{\#}$ ** (789.72, 26.33)	673.89 ± 37.95 ${\;}^{*}$ (665.69, 64.20)	a = 0.01 b = 0.01 c = 0.85 d ≤ 0.001 e = 0.04 f ≤ 0.001
**Preantral follicles**	355.75 ± 17.28 ${\;}^{*}$ (357.81, 29.68)	301.69 ± 28.83 ** ${\;}^{†}$ ** (308.98, 46.42)	401.20 ± 20.35 ** ${\;}^{\#}$ ** (403.66, 35.91)	338.31 ± 22.08 ${\;}^{*}$ (328.73, 40.34)	a = 0.01 b = 0.01 c = 0.55 d ≤ 0.001 e = 0.04 f = 0.01
**Antral follicles**	174.91 ± 14.01 ${\;}^{*}$ (175.33, 19.56)	145.65 ± 13.21 ** ${\;}^{†}$ ** (150.95, 19.90)	201.38 ± 6.60 ** ${\;}^{\#}$ ** (202.29, 12.60)	169.27 ± 18.59 ${\;}^{*}$ (169.39, 28.83)	a = 0.01 b = 0.02 c = 0.89 d ≤ 0.001 e = 0.04 f = 0.01
Data presented as Mean ± SD (MD, IQR). ANOVA and Tukey's post hoc test. The superscripts (*, † , and #) show significant differences within means at p < 0.05 in the same row. The different scripts show p-value within means in each row. a: Control vs AgNPs, b: Control vs TQ, c: Control vs TQ + AgNPs, d: AgNPs vs TQ, e: AgNPs vs TQ + AgNPs, f: TQ + AgNPs vs TQ, TQ: Thymoquinone, AgNPs: Silver nanoparticles					

## 4. Discussion

This study aimed to analyze the effects of TQ as a potential natural antioxidant on AgNPs-induced ovarian tissue toxicity, focusing on stereological and biochemical effects. In the present study, the administration of AgNPs led to a reduction in the TAC and increased lipid peroxidation, both of which resulted in a decrease in the number of follicles (primordial, primary, preantral, and antral) as well as the volume of the corpus luteum. In addition, according to our research, AgNPs reduced the levels of LH and FSH. Our experiment showed that in the co-administration group (TQ + AgNPs), TQ caused a significant increase in the number of primary, preantral, and antral follicles by improving the TAC, FSH, and LH levels and reducing the lipid peroxidation. However, TQ co-administration did not significantly increase the number of primordial follicles compared to the AgNPs group. Despite these results, no significant difference was observed in the thickness of the zona pellucida in the preantral and antral follicles, as well as the volume of the oocyte and its nucleus. In summary, based on the in vivo and in vitro studies, the toxic effects of AgNPs on ovarian tissue undergo significant changes depending on the dose, size, and exposure time (4, 7, 19). The intraperitoneal injection of AgNPs (at doses of 0.5, 1, and 5 for 35 days) caused oxidative stress and lipid peroxidation. AgNPs disrupted the Cyp19a1 (cytochrome P450, family 19, subfamily a, polypeptide 1) activity, reducing preantral and antral follicles via decreased estradiol and angiogenesis (12). A previous study has reported that the injection of a single dose of AgNPs (0.5, 1 mg/kg) caused a decrease in the expression of mRNA involved in the growth and survival of follicles such as forkhead box O3, and folliculogenesis-specific basic helix-loop-helix transcription factor. It also induces mitochondrial apoptosis damage to granulosa cells and causes a decrease in the level of estrogen by disrupting the activities of Cyp11a1 and Cyp17a1 enzymes (10). Recent findings demonstrated that AgNPs led to a decrease in estrogen levels, changes in sex hormone levels, and an increase in the expression of caspase 3; these alterations trigger the degeneration of follicles, hyperplasia, and apoptosis in uterine epithelial cells (20). Similarly, several studies also confirm our findings (4, 21). However, nanoparticles can pass through the blood-brain barrier and subsequently change the levels of FSH, LH, estrogen, and progesterone by disrupting the hypothalamic-pituitary-gonadal axis; no reports were found about the penetration of AgNPs into the oocyte. The layers surrounding the follicles and zona pellucida probably do not allow nanoparticles to enter the cytoplasm. Hence, these nanoparticles accumulate in granulosa and theca cells, and by damaging these cells, AgNPs induce apoptosis in follicular cells (22). TQ balances FSH, LH, and testosterone in polycystic ovary syndrome. It also promoted the expression of the glutathione peroxidase gene, the growth of primordial, primary, preantral, and antral follicles, and improved corpus luteum volume. Additionally, TQ prevented apoptosis of follicle cells in the ovarian tissue by inhibiting the expression of B-cell lymphoma 2 (Bcl-2)-associated X protein as an apoptotic activator and increasing the expression of Bcl-2 as an apoptotic inhibitor (17). Our finding showed no significant changes in the total volume of the ovary, medulla, and cortex between experimental groups. Exposure to AgNPs with vaginal gel reduced reproductive ability due to the toxic effect on blastocysts and the early development of embryos in female mice. However, AgNPs do not change ovarian tissue (11).

In addition, previous studies have shown that TQ improves folliculogenesis and regulates hormone levels by improving the level of TAC and catalase and inhibiting the inflammatory factor cyclooxygenase 2 against ovarian tissue toxicity induced by the carcinogen acrylamide (23). TQ upregulated the aromatase level, reduced inflammatory cytokines, and autophagic markers, simulating super-ovulated condition in polycystic ovary syndrome (24). Other studies have demonstrated that TQ increases ovarian tissue cell survival by upregulating Bcl-2, downregulating Bcl-2-associated X protein, and reducing caspase 3 in response to toxicities (25). According to these studies, our findings were consistent with previous reports.

Our data did not show any change in body and ovary weight. The administering of AgNPs (4 mg/kg for 30 days) to female rats did not affect the ovaries and body weight (20). Our results confirm previous studies that exposure to AgNPs in a short time does not significantly affect changes in food metabolism and weight loss or gain.

Overall, based on previous research and our findings, considering that AgNPs in the ovary induce mitochondrial apoptosis and oxidative stress, we inferred that TQ effectively reverses apoptosis of ovarian follicles by inhibiting apoptosis and oxidative stress and regulating the hormonal and cytokines levels.

## 5. Conclusion

Our findings showed that AgNPs caused ovarian lipid peroxidation and oxidative stress and reduced the hormones, number of follicles, and corpus luteum volume. TQ co-administration reversed these effects, increasing the hormone levels, the number of primary, preantral, and antral follicles, and corpus luteum volume.

##  Data availability

Data supporting the findings of this study are available upon reasonable request from the corresponding author.

##  Author contributions

Seyed Mohammad Ali Shariatzadeh had full access to all of the data in the study and takes responsibility for the integrity of the data and the accuracy of the data analysis. Concept and design: Seyed Mohammad Ali Shariatzadeh, Fatemeh Salmani, and Monireh Mahmoodi. Acquisition, analysis, or interpretation of data: Fatemeh Salmani, Hossein Moghanlo, and Monireh Mahmoodi. Drafting of the manuscript: Hossein Moghanlo and Fatemeh Salmani. Critical revision of the manuscript for important intellectual content: All authors. Final approval of the version to be published: All authors. Statistical analysis: Fatemeh Salmani and Hossein Moghanlo. Supervision: Seyed Mohammad Ali Shariatzadeh.

##  Conflict of Interest

The authors state that there is no conﬂict of interest.

## References

[bib1] Bala R, Singh V, Rajender S, Singh K (2021). Environment, lifestyle, and female infertility. Reprod Sci.

[bib2] Liao Ch, Li Y, Tjong SC (2019). Bactericidal and cytotoxic properties of silver nanoparticles. Int J Mol Sci.

[bib3] Burdușel A-C, Gherasim O, Grumezescu AM, Mogoantă L, Ficai A, Andronescu E (2018). Biomedical applications of silver nanoparticles: An up-to-date overview. Nanomaterials.

[bib4] Li Y, Cummins E (2020). Hazard characterization of silver nanoparticles for human exposure routes. J Environment Sci Health Part A.

[bib5] Moghanlo H, Shariatzadeh SMA (2022). Beneficial effects of Spirulina platensis on mice testis damaged by silver nanoparticles. Andrologia.

[bib6] Zhang J, Wang F, Yalamarty SSK, Filipczak N, Jin Y, Li X (2022). Nano silver-induced toxicity and associated mechanisms. Int J Nanomed.

[bib7] Wang R, Song B, Wu J, Zhang Y, Chen A, Shao L (2018). Potential adverse effects of nanoparticles on the reproductive system. Int J Nanomed.

[bib8] Santacruz-Márquez R, Gonzalez-De los Santos M, Hernandez-Ochoa I (2021). Ovarian toxicity of nanoparticles. Reprod Toxicol.

[bib9] Zhang J, Liu S, Han J, Wang Z, Zhang S (2021). On the developmental toxicity of silver nanoparticles. Mater Des.

[bib10] Han JW, Jeong J-K, Gurunathan S, Choi Y-J, Das J, Kwon D-N, et al (2016). Male-and female-derived somatic and germ cell-specific toxicity of silver nanoparticles in mouse. Nanotoxicology.

[bib11] Zhang D, Yu F, Li H, Wang Q, Wang M, Qian H, et al (2022). AgNPs reduce reproductive capability of female mouse for their toxic effects on mouse early embryo development. Hum Exp Toxicol.

[bib12] Mirzaei M, Razi M, Sadrkhanlou R (2017). Nanosilver particles increase follicular atresia: Correlation with oxidative stress and aromatization. Environment Toxicol.

[bib13] Hannan MA, Rahman MA, Sohag AAM, Uddin MJ, Dash R, Sikder MH, et al (2021). Black cumin (Nigella sativa L. ): A comprehensive review on phytochemistry, health benefits, molecular pharmacology, and safety Nutrients.

[bib14] Sadeghi E, Imenshahidi M, Hosseinzadeh H (2023). Molecular mechanisms and signaling pathways of black cumin (Nigella sativa) and its active constituent, thymoquinone: A review. Mol Biol Rep.

[bib15] Sarkar C, Jamaddar S, Islam T, Mondal M, Islam MT, Mubarak MS (2021). Therapeutic perspectives of the black cumin component thymoquinone: A review. Food Function.

[bib16] Darakhshan S, Pour AB, Colagar AH, Sisakhtnezhad S (2015). Thymoquinone and its therapeutic potentials. Pharmacol Res.

[bib17] Alaee S, Mirani M, Derakhshan Z, Koohpeyma F, Bakhtari A (2023). Thymoquinone improves folliculogenesis, sexual hormones, gene expression of apoptotic markers and antioxidant enzymes in polycystic ovary syndrome rat model. Vet Med Sci.

[bib18] Sanamiri K, Mehranjani MS, Shahhoseini M, Shariatzadeh MA (2022). L-carnitine improves follicular survival and function in ovarian grafts in the mouse. Reprod Fertil Dev.

[bib19] Rezvani E, Rafferty A, McGuinness C, Kennedy J (2019). Adverse effects of nanosilver on human health and the environment. Acta Biomater.

[bib20] Mohamed Y, El Ghareeb A-W, Attaby FA, Abd El-Rahman HA (2022). Estimation of silver nanoparticles effect on the reproductive health of female Wistar rats. Egypt J Basic Appl Sci.

[bib21] Pourali P, Nouri M, Ameri F, Heidari T, Kheirkhahan N, Arabzadeh S, et al (2020). Histopathological study of the maternal exposure to the biologically produced silver nanoparticles on different organs of the offspring. Naunyn Schmiedebergs Arch Pharmacol.

[bib22] Hou C-C, Zhu J-Q (2017). Nanoparticles and female reproductive system: How do nanoparticles affect oogenesis and embryonic development. Oncotarget.

[bib23] AL-ghamdi M, Huwait E, Elsawi N, Ali SS, Sayed A (2023). Thymoquinone ameliorates acrylamide-induced reproductive toxicity in female rats: An experimental study. Int J Reprod BioMed.

[bib24] Saha P, Kumar S, Datta K, Tyagi RK (2021). Upsurge in autophagy, associated with mifepristone-treated polycystic ovarian condition, is reversed upon thymoquinone treatment. J Steroid Biochem Mol Biol.

[bib25] Eini F, Bidadkosh A, Nazarian H, Piryaei A, Ghaffari Novin M, Joharchi K (2019). Thymoquinone reduces intracytoplasmic oxidative stress and improves epigenetic modification in polycystic ovary syndrome mice oocytes, during in‐vitro maturation. Mol Reprod Dev.

